# A decision tree based on procalcitonin and C-reactive protein levels as a potential diagnostic tool to distinguish PFAPA flares from acute bacterial and viral infections

**DOI:** 10.1186/1546-0096-13-S1-P167

**Published:** 2015-09-28

**Authors:** B Kraszewska-Glomba, Z Szymanska-Toczek, L Szenborn

**Affiliations:** 1 Wroclaw Medical University, Department and Clinic of Pediatric and Infectious Diseases, Wroclaw, Poland

## Introduction

Periodic fever, aphthous stomatitis, pharyngitis and cervical adenitis (PFAPA) is a disease of unknown etiology and unclear pathophysiology. Considering the inexistence of specific laboratory test for PFAPA, it remains a diagnosis of exclusion.

## Objective

We searched for practical use of procalcitonin (PCT) and C-reactive protein (CRP) in differentiating PFAPA attacks from acute bacterial and viral infections.

## Methods

Levels of PCT and CRP were measured in 35 PFAPA patients during 67 PFAPA febrile episodes and in 86 children diagnosed with acute bacterial (n=47) or viral (n=39) infection. We used the C4.5 algorithm (statistical classifier) to construct a decision tree.

## Results

Statistical analysis with the use of C4.5 algorithm resulted in the following decision tree: viral infection if CRP≤19.1 mg/L; otherwise for cases with CRP>19.1 mg/L: PFAPA if PCT≤0.65 ng/mL, bacterial infection if PCT>0.65ng/mL. The rule was effective in 83.7% of the cases. Febrile episodes during PFAPA flares, bacterial and viral infections were classified with the sensitivity of 76.1%, 93.6% and 84.6% and specificity of 89.5%, 88.7% and 96.5% respectively.

## Conclusion

Differences in PCT and CRP levels during PFAPA attacks, bacterial and viral diseases may be used to build a simple decision tree. When interpreted cautiously and with reference to the clinical context, it might present a potential diagnostic tool for distinguishing PFAPA flares from acute infections.

